# Amorphous pyrano[3,2-c]quinoline-semicarbazone thin films switch from uniform to heterogeneous morphology and optical modulation upon copper coordination

**DOI:** 10.1038/s41598-026-69613-0

**Published:** 2026-09-15

**Authors:** A. A. El-Saady, Magdy A. Ibrahim, A. M. Mansour, M. M. El-Nahass, Omima M.I. Adly, A. A.M. Farag

**Affiliations:** 1 Thin Films Laboratory, Physics Department, Faculty of Education, Ain Shams University, Roxy, 11757 Cairo Egypt; 2 Department of Chemistry, Faculty of Education, Ain Shams University, Roxy, 11757 Cairo Egypt; 3 Solid State Physics Department, Physics Research Institute, National Research Centre, Dokki, 12622 Giza Egypt

**Keywords:** Pyranoquinoline-semicarbazone (PQMHC), Cu(II) complex thin films, Thermal evaporation, Photoluminescence, Optical properties, Materials science, Nanoscience and technology, Optics and photonics, Physics

## Abstract

Thermally evaporated pyrano[3,2-c]quinoline semicarbazone (**PQMHC**) and its Cu(II) complex thin films were systematically investigated to elucidate the effect of metal coordination on their structural, morphological, photoluminescence, and optical properties. X-ray diffraction (XRD) revealed broad halos, confirming the amorphous nature of both films. Field-emission scanning electron microscopy (FE-SEM) showed that the **PQMHC** film possesses a relatively uniform surface composed of closely packed granular features, whereas Cu(II) incorporation induces a heterogeneous morphology with irregular clusters and elongated structures. Photoluminescence measurements revealed broad visible emission spanning 380–650 nm, with a pronounced red shift toward 520–560 nm upon Cu(II) complexation. Optical measurements showed reduced transmittance and enhanced reflectance following Cu(II) incorporation, accompanied by a decrease in the optical band gap from 2.87 ± 0.02 to 2.57 ± 0.01 eV. Furthermore, Miller’s empirical rule was employed to estimate the third-order nonlinear susceptibility and nonlinear refractive index, both of which decreased upon Cu(II) coordination, indicating reduced calculated polarizability. Overall, Cu(II) coordination provides an effective means of tuning the structural, morphological, photoluminescence, and optical characteristics of **PQMHC** thin films, suggesting their potential for tunable optical and optoelectronic applications.

## Introduction

Organic and metal–organic thin films have emerged as key materials for next-generation optoelectronic and photonic applications due to their tunable structural, morphological, and optical properties. In particular, π-conjugated heterocyclic systems exhibit light–matter interaction, making them promising for applications such as optical switching, photodetectors, and nonlinear optical (NLO) devices. Recent studies have emphasized that tailoring molecular structure and intermolecular interactions significantly enhances optical responses, especially in hybrid organic–inorganic systems^1^.

Among these materials, semicarbazone-based compounds have gained increasing attention owing to their versatile coordination behavior, structural flexibility, and efficient charge-transfer characteristics. The incorporation of pyranoquinoline moieties further enhances π-electron delocalization, resulting in improved optical absorption and emission properties. Recent reports indicate that pyranoquinoline derivatives exhibit direct optical band gaps in the range of 2–3 eV, along with fluorescence and high optoelectronic performance, suggesting possible potential for future photonic and nonlinear optical investigations subject to direct experimental validation and energy-related applications^2,3^.

The coordination of transition metal ions, particularly Cu(II), with semicarbazone ligands introduces significant modifications in structural and electronic properties. Metal–ligand interaction leads to the redistribution of electron density, the modification of frontier molecular orbitals, and the enhancement of charge-transfer transitions. Recent experimental and theoretical investigations have demonstrated that Cu(II)–semicarbazone complexes exhibit improved stability, altered band structure, and enhanced optical activity compared to their parent ligands^4,5^. Furthermore, the chelation process often results in rigid molecular frameworks and increased conjugation, which directly influence optical performance.

In addition to linear optical properties, nonlinear optical behavior has become a major focus due to its importance in optical limiting, all-optical switching, and laser technologies. Recent studies have confirmed that Cu-based coordination complexes exhibit enhanced third-order nonlinear susceptibility due to ligand-to-metal charge transfer (LMCT), defect states, and improved carrier mobility^6,7^. Similarly, transition metal complexes derived from Schiff base and hydrazone ligands have demonstrated strong NLO responses, attributed to reduced HOMO–LUMO gaps and increased electronic polarization^8,9^. These findings highlight the critical role of metal incorporation in tuning nonlinear optical properties.

In recent years, increasing attention has been directed toward organic and metal-organic thin films exhibiting strong nonlinear optical (NLO) response and tunable optoelectronic characteristics. They have potential applications in optical limiting, ultrafast switching, waveguiding, and photonic modulation devices. Previous investigations demonstrated that charge-transfer interactions, dielectric relaxation behavior, and refractive index modulation strongly influence third-order nonlinear susceptibility and optical conductivity in coordination-based thin films^10–12^. In particular, Cu-containing systems have shown enhanced light-matter interaction, improved carrier transport, and tunable optical dispersion arising from metal-ligand hybridization and electronic delocalization. Recent studies also emphasized that morphological disorder and localized electronic states significantly affect nonlinear optical performance and dielectric response in amorphous and semi-amorphous thin-film systems^6–9^. Therefore, establishing a direct correlation between structural disorder, morphology evolution, and nonlinear optical behavior remains essential for the rational design of advanced optoelectronic materials.

Moreover, recent integrated studies have emphasized the importance of correlating experimental and theoretical approaches to fully understand structure–property relationships. Density functional theory (DFT) calculations, combined with spectroscopic techniques, have been successfully used to explain electronic structure, charge distribution, and NLO behavior in Cu(II) complexes and related systems^13^. Despite these advances, comprehensive studies that simultaneously correlate structural (XRD), morphological (FE-SEM), photoluminescence, and both linear and nonlinear optical properties in semicarbazone pyranoquinoline systems remain limited.

Despite the rapid progress in organic and metal-organic thin films for optoelectronic and photonic applications, several important scientific gaps remain unresolved. Most previous studies have primarily focused on isolated optical measurements or limited structural characterization without providing an integrated understanding of how morphology, structural disorder, dielectric response, and nonlinear optical properties collectively govern thin-film performance. In particular, semicarbazone-based pyranoquinoline systems have received limited attention despite their conjugated framework and charge-transfer capability, which are desirable for nonlinear photonic applications. Moreover, although Cu(II) coordination is known to modify electronic structure and optical activity, experimental studies addressing its influence on thin-film nonlinear susceptibility, refractive index dispersion, optical conductivity, and dielectric relaxation remain scarce. Existing reports also lack comprehensive correlations between amorphous morphology, localized electronic states, optical band-gap modulation, and nonlinear optical response within a unified experimental framework. Consequently, a systematic investigation linking structural, morphological, linear optical, dielectric, and nonlinear optical properties is still required to fully understand and optimize these materials for advanced optoelectronic and photonic applications.

Transition-metal coordination compounds have attracted considerable attention as multifunctional materials for optoelectronic, photonic, sensing, and nonlinear-optical applications because their electronic structures and optical responses can be tailored through metal–ligand interactions. Shatir et al.^1^ have investigated the linear, nonlinear optical, and dielectric properties of transition-metal complex films derived from an azo-Schiff base, demonstrating the importance of molecular structure and metal coordination in controlling their optical and dielectric characteristics. El-Saady et al.^2^ have reported the structural, morphological, and optical modifications of semicarbazone pyranoquinoline (**PQMHC**) and its Cu(II) complex, revealing the influence of Cu(II) coordination on their properties and photocatalytic performance. However, that study was primarily concerned with powder/nanofibrous forms and diffuse-reflectance-based optical analysis. Abdel-Megid et al.^3^ have investigated a Cu(II)-pyrano[3,2-c]quinoline complex through DFT-assisted spectroscopic and nonlinear-optical/NBO analyses, while El-Saady et al.^13^ have reported the synthesis, spectroscopic characterization, and DFT-based structural insights of a Cu(II) semicarbazone–pyranoquinoline complex. According to these studies, the structural and molecular characteristics of the Cu(II)–**PQMHC** system have been established; however, its structure–property relationships in the thermally evaporated thin-film state remain unexplored.

Recent studies have highlighted the versatility of Cu-containing materials and functional thin films for sensing, optoelectronic, and photonic applications. Roh et al.^4^ demonstrated Cu-based conductive metal–organic framework thin films for ultrasensitive chemical detection, while Özel and Demirezen^5^ reported Cu-containing hybrid photodiodes with potential for photonic and electronic applications. In the nonlinear-optical domain, Manurkar et al.^6^ established the functional optical response of Cu(II) coordination complexes, and Thundiyil et al.^7^ demonstrated that Cu incorporation can modulate the nonlinear optical response of NiO thin films. Complementary studies by Hussain et al.^8^ and Tahmasbi et al.^9^ further confirmed the importance of Cu(II) coordination in tailoring nonlinear optical properties. Beyond molecular and coordination systems, Shiau et al.^14^ demonstrated enhanced UV photodetector performance in MgZnO thin films, whereas Lee et al.^15^ showed that compositional modification through Ga doping and Mg alloying can strongly influence the stress-sensitive response of MgZnO-based films. Moreover, Giri et al.^10^, Mohanty et al.^11^, and Das et al.^12^ established that structural evolution and thermal processing can substantially modify the optical, electrical, morphological, and nonlinear optical characteristics of thin-film materials. Collectively, these studies underscore the critical roles of composition, metal incorporation, structural evolution, and morphology in governing the functional properties of advanced thin films. However, despite these advances, the combined influence of Cu(II) coordination on the structural, morphological, photoluminescence, linear-optical, and nonlinear-optical properties of thermally evaporated **PQMHC**-based thin films remains unexplored.

The novelty of the present work lies in extending the Cu(II)–**PQMHC** system from previously investigated molecular, powder/nanofibrous, and theoretical platforms^2,3,13^ to thermally evaporated thin films and, importantly, in establishing a unified structure–morphology–optical framework for both the ligand and its Cu(II) complex. Unlike our previous studies, which focused on structural, spectroscopic, photocatalytic, electrochemical-sensing, and DFT-based molecular properties^2,3,13^, the present investigation directly determines the thin-film optical response from transmittance and reflectance measurements. This enables quantitative evaluation of the refractive index (n), extinction coefficient (k), dielectric constants (ε₁ and ε₂), optical band gap, Wemple–DiDomenico dispersion parameters, electric modulus, volume and surface energy-loss functions, optical conductivity, and third-order nonlinear optical parameters based on Miller’s rule. These measurements are further correlated with structural characteristics, surface morphology, and photoluminescence behavior, thereby providing new insight into the effects of Cu(II) coordination on electronic transitions, optical dispersion, emission processes, energy-loss mechanisms, and nonlinear optical functionality. The present study therefore represents more than a change in sample form; it establishes a previously unavailable experimental structure–property relationship for this ligand–complex pair in the thin-film state.

The main objective of this study is to quantitatively and qualitatively investigate the impact of Cu(II) incorporation on **PQMHC** thin films by correlating their structural characteristics, morphological evolution, photoluminescence behavior, and linear and nonlinear optical parameters. Specifically, the study aims to determine how metal–ligand interaction modifies the band-gap energy, optical constants, emission characteristics, and third-order nonlinear susceptibility. By integrating these complementary measurements within a single experimental framework, the study seeks to elucidate the fundamental relationship between Cu(II) coordination, structural and morphological modification, and the resulting optical functionality. This objective provides a coherent basis for interpreting the structure–property relationships and for assessing the potential of **PQMHC** and its Cu(II) complex as functional materials for advanced photonic and optoelectronic applications.

## Experimental techniques

The 2-[(6-ethyl-4-hydroxy-2,5-dioxo-5,6-dihydro-2 H-pyrano[3,2-c]quinolin-3-yl)methylidene] hydrazinecarboxamide **(PQMHC)** ligand and its Cu(II) complex powders utilized in this study were synthesized following the procedure reported in our previous work^16^. Thin films were deposited onto thoroughly cleaned glass and quartz substrates via thermal evaporation under a high vacuum of $\:1.5\times\:{10}^{-5}$ Torr is using an Edwards E306 coating system. Before deposition, the substrates were sequentially cleaned with detergent, deionized water, acetone, and ethanol, followed by ultrasonic treatment to ensure complete removal of contaminants and improved surface uniformity.

Deposition was carried out at room temperature (300 K) using a molybdenum boat to ensure stable evaporation and to minimize contamination. The film thickness was monitored in situ during deposition using a calibrated quartz thickness monitor integrated into the thermal evaporation system. Before deposition, the monitor was calibrated with a standard reference to ensure accurate thickness control. The deposition rate was maintained at approximately 2.5 Å s⁻¹, yielding films with a nominal thickness of about 300 nm.

The structural characteristics of the **PQMHC** ligand and its Cu(II) complex thin films were investigated by X-ray diffraction (XRD) using a Bruker D8 ADVANCE diffractometer equipped with CuK$\:\alpha\:$ radiation ($\:\lambda\:=0.154060$ nm). The instrument was operated at an accelerating voltage of 40 kV and a tube current of 40 mA. Diffraction patterns were recorded over a $\:2\theta\:$ range of 5°–80°, with a step size of 0.05° and a scanning rate of 2°$\:/\mathrm{m}\mathrm{i}\mathrm{n}\:$.

The surface morphology of the prepared thin films was examined using field emission scanning electron microscopy (FE-SEM) (Quanta FEG 250). An accelerating voltage of 30 kV was applied to obtain high-resolution surface images. Representative micrographs were collected from multiple regions of each sample to confirm surface homogeneity and reproducibility of morphological features.

The photoluminescence (PL) properties of the **PQMHC** ligand and its Cu(II) complex were investigated at room temperature using a Jasco FP-6500 spectrofluorometer equipped with a 150 W xenon flash lamp as the excitation source. A slit width of 5 nm was employed to achieve high spectral resolution. Excitation was performed at a wavelength of 350 nm, and emission spectra were recorded over the range of 360–900 nm with a scan step of 1 nm.

The optical transmittance (T) and reflectance (R) spectra of the as-deposited thin films were measured using a double-beam spectrophotometer (JASCO V-670 UV–Vis–NIR) over the wavelength range of 200–2500 nm. Baseline correction and instrument calibration were performed prior to measurements using standard reference materials.

To ensure experimental reproducibility, all thin-film preparation and characterization measurements were performed under identical and controlled experimental conditions, as described in the Experimental Section. Independent measurements were repeated for each sample, and the reported quantitative parameters are presented as mean ± standard deviation (SD). The SD values were calculated from the repeated measurements to quantify the experimental variability and measurement uncertainty. The obtained results showed low standard deviations relative to the corresponding mean values, confirming good experimental repeatability. Importantly, the observed differences between the **PQMHC** and Cu(II)-**PQMHC** thin films were substantially larger than the associated experimental uncertainties, supporting the reliability and reproducibility of the reported structure–property relationships.

### Theoretical considerations

The measured transmittance and reflectance data were carefully corrected to eliminate the contribution of the quartz substrate, ensuring that the extracted optical response represents only the intrinsic properties of the deposited films. The correction was performed using the relations proposed by Zeyada et al.^17^:

1 $$\:T=\left(\frac{{I}_{ft}}{{I}_{q}}\right)\left(1-{R}_{q}\right)$$

2 $$\:R=\left(\frac{{I}_{fr}}{{I}_{m}}\right){R}_{m}\left[{\left(1-{R}_{q}\right)}^{2}+1\right]-{T}^{2}{R}_{q}$$

where $\:{I}_{ft}$ denotes the transmitted intensity through the quartz film system, $\:{I}_{q}$ is the transmitted intensity through the reference quartz, $\:{R}_{q}$ represents the reflectance of quartz, $\:{I}_{fr}$ denotes the reflected intensity from the sample, $\:{I}_{m}$ corresponds to the reflected intensity from the reference Al mirror, and $\:{R}_{m}$ represents the mirror reflectance.

The absorption coefficient $\:\alpha\:$ was calculated from the absolute transmittance * T*, reflectance * R*, and film thickness * d* using^18^:

3 $$\:\alpha\:=\frac{1}{d}{ln}\left[\frac{{\left(1-R\right)}^{2}}{2T}+\sqrt{\frac{{\left(1-R\right)}^{4}}{4{T}^{2}}+{R}^{2}}\:\right]$$

The extinction coefficient * k* was determined from^19^:

4 $$\:k=\frac{\alpha\:\lambda\:}{4\pi\:}$$

The absorption coefficient follows the Tauc relation^20^:

5 $$\:\left(\alpha\:h\nu\:\right)=G(h\nu\:-{E}_{g}{)}^{r}$$

where * G* is a proportionality constant associated with optical absorption and the probability of electronic transitions, $\:h\nu\:$ denotes the photon energy, and * r* depends on the nature of the transition (* r=1/2* for allowed direct and * r=2* for allowed indirect transitions)^21^.

The refractive index * n* was evaluated from reflectance data using^22^:

6 $$\:n=\frac{1+R}{1-R}+\sqrt{\frac{4R}{{\left(1-R\right)}^{2}}-{k}^{2}}$$

To analyze dispersion, the refractive index was fitted using the Wemple–DiDomenico single-oscillator model^23,24^:

7 $$\:{({n}^{2}-1)}^{-1}=\frac{{E}_{o}}{{E}_{d}}-\frac{{\left(h\nu\:\right)}^{2}}{{E}_{o}{E}_{d}}$$

where $\:{E}_{o}$ denotes the oscillator energy and $\:{E}_{d}$ represents the dispersion energy, representing the strength of interband optical transitions.

The refractive index can also be expressed as a function of wavelength as follows^25^:

8 $$ \:n^{2} = \varepsilon \:_{L} - \frac{{e^{2} N}}{{4\pi \:^{2} \varepsilon \:_{o} m^{*} c^{2} }}\lambda \:^{2} $$

where $\:{ \varepsilon\:}_{L}$ denotes the lattice dielectric constant, * e* is the elementary charge, * c* the speed of light, $\:{ \varepsilon\:}_{0}$ corresponds to the permittivity of free space, and $\:N/{m}^{*}$ represents the carrier concentration to effective mass ratio.

The complex dielectric constant $\:{ \varepsilon\:}^{*}={ \varepsilon\:}_{1}+i{ \varepsilon\:}_{2}$ is related to * n* and * k* by^26^:

9 $$\:{ \varepsilon \:}_{1}={n}^{2}-{k}^{2}$$

10 $$\:{ \varepsilon\:}_{2}=2nk$$

The complex electric modulus $\:{M}^{*}={M}_{1}+i{M}_{2}$ is defined as the inverse of the complex dielectric constant and evaluated using^27^:

11 $$\:{M}_{1}=\frac{{ \varepsilon \:}_{1}}{{ \varepsilon\:}_{1}^{2}+{\epsilon\:}_{2}^{2}}$$

12 $$\:{M}_{2}=\frac{{\varepsilon\:}_{2}}{{\varepsilon\:}_{1}^{2}+{\varepsilon\:}_{2}^{2}}$$

The volume energy loss function (VELF) and the surface energy loss function (SELF) are given by^28^:

13 $$\:\mathrm{VELF}=\frac{{ \varepsilon \:}_{2}}{{\varepsilon\:}_{1}^{2}+{ \varepsilon\:}_{2}^{2}}$$

14 $$\:\mathrm{SELF}=\frac{{\varepsilon\:}_{2}}{\left({\varepsilon\:}_{1}+1{)}^{2}+{\varepsilon\:}_{2}^{2}\right.}$$

The complex optical conductivity $\:{\sigma\:}^{*}={\sigma\:}_{1}+i{\sigma\:}_{2}$ is expressed as^29^:

15 $$\:{\sigma\:}_{1}=\omega\:{\varepsilon\:}_{0}{\varepsilon\:}_{2}$$

16 $$\:{\sigma\:}_{2}=\omega\:{\varepsilon\:}_{0}{\varepsilon\:}_{1}$$

where $\:\omega\:$ corresponds to the angular frequency.

According to Miller’s rule, the linear susceptibility $\:{\chi\:}^{\left(1\right)}$ is given by^30^:

17 $$\:{\chi\:}^{\left(1\right)}=\frac{{n}_{0}^{2}-1}{4\pi\:}$$

where $\:{n}_{0}$ denotes the static refractive index. The third-order nonlinear susceptibility $\:{\chi\:}^{\left(3\right)}$ is expressed as^31^:

18 $$\:{\chi\:}^{\left(3\right)}=A{\left[{\chi\:}^{\left(1\right)}\right]}^{4}$$

where $\:A\approx\:1.7\times\:{10}^{-10}{\hspace{0.17em}}\mathrm{esu}$. The nonlinear refractive index $\:{n}_{2}$ is given by^32^:

19 $$\:{n}_{2}=\frac{12\pi\:{\chi\:}^{\left(3\right)}}{{n}_{0}}$$

Finally, the optical electronegativity $\:{\chi\:}_{\mathrm{opt}}$ is estimated as^33^:

20 $$\:{\chi\:}_{\mathrm{opt}}={\left(\frac{25.54}{{n}_{0}}\right)}^{1/4}$$

## Results and discussion

### Structural analysis

#### X‑ray diffraction (XRD) analysis

**Fig. 1:**
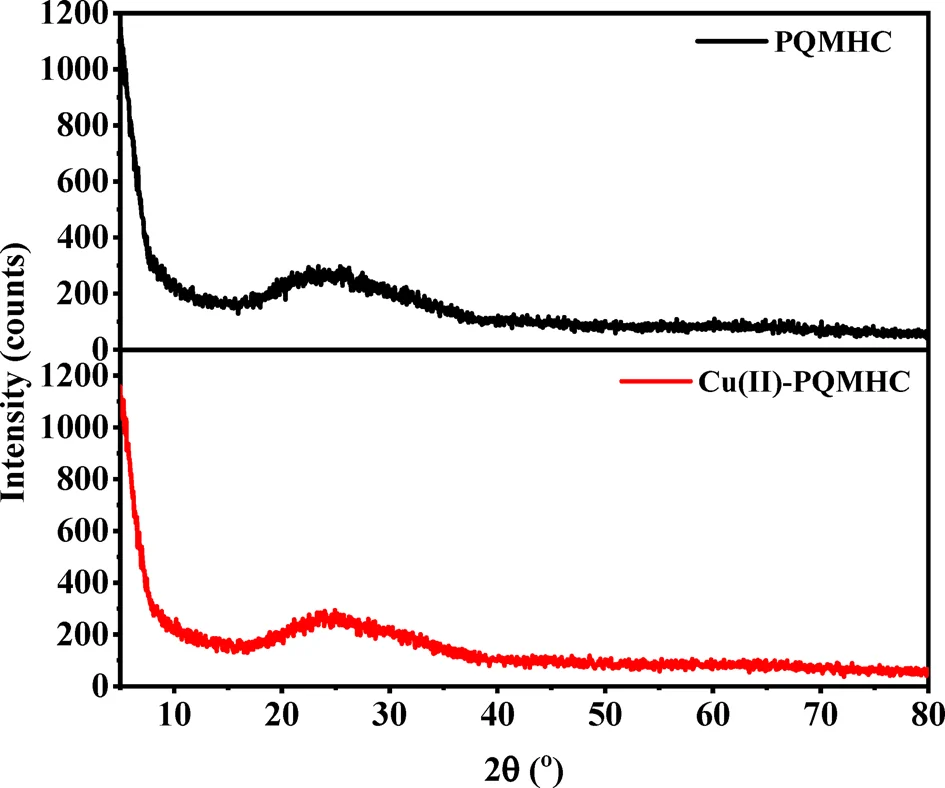
XRD pattern of **PQMHC** ligand and its Cu(II) complex thin films.

The X ray diffraction patterns of the **PQMHC** ligand and its Cu(II) complex thin films (Fig. 1) exhibit a broad diffuse halo without distinct Bragg reflections confirming their predominantly amorphous nature This behavior indicates the absence of long range periodicity and reflects a structurally disordered arrangement governed by short range molecular correlations A slight shift in the halo position upon Cu(II) complexation reveals a modification in the average intermolecular spacing while the observed change in peak broadening suggests a variation in the correlation length These features provide clear evidence that metal coordination induces significant rearrangement in molecular packing and alters the local structural organization within the thin films Although crystallinity is not observed the amorphous nature of these films offers distinct advantages for optoelectronic applications The isotropic structure ensures uniform optical response across the film surface while supporting large area deposition on flexible substrates In addition the tunability of the electronic structure in amorphous systems enables effective control of the optical band gap and electrical properties thereby enhancing their suitability for advanced device engineering^34^.

#### Field emission scanning electron microscope (FE-SEM) analysis

**Fig. 2:**
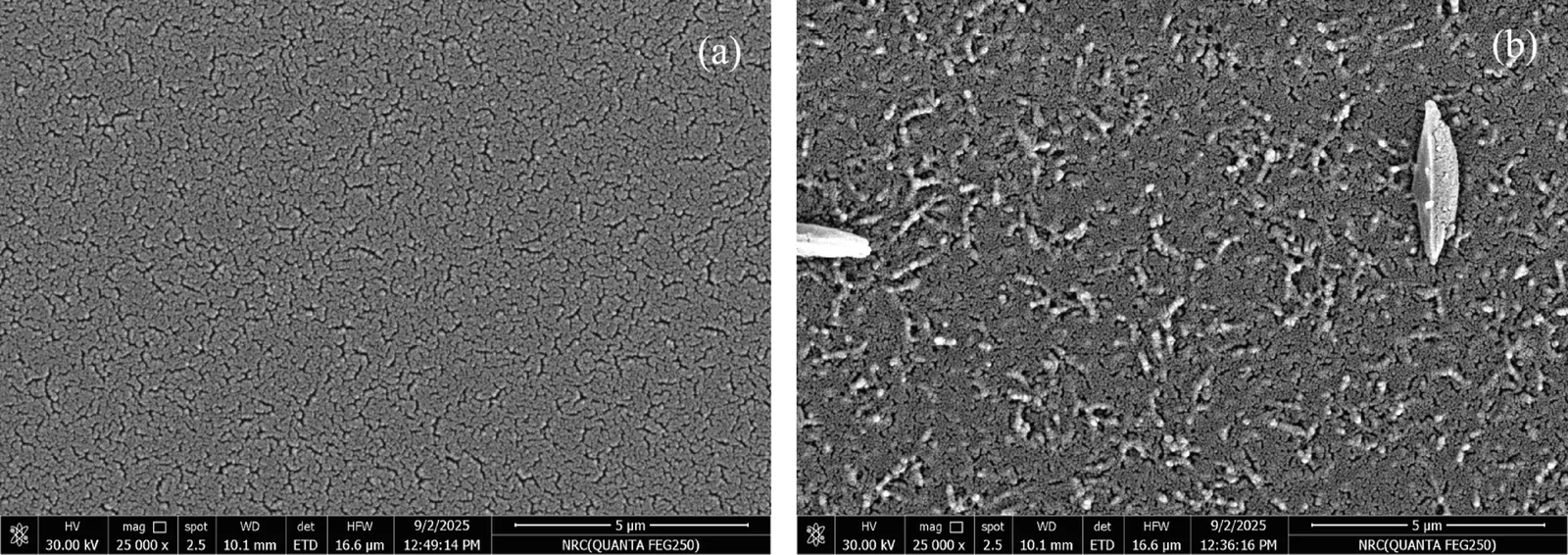
FE-SEM micrographs of magnification 25 000 × for (**a**) **PQMHC** ligand, and (**b**) its Cu(II) complex thin films.

The FE-SEM micrographs recorded at 25,000× magnification (Fig. 2(a, b)) reveal a pronounced Cu(II)-induced modification of the surface morphology of the **PQMHC**-based thin films. The pristine **PQMHC** film (Fig. 2(a)) exhibits a relatively uniform and compact surface consisting of fine, closely distributed granular features with an estimated characteristic lateral dimension of approximately 0.2–0.6 μm, as determined from the calibrated 5 μm scale bar. The relatively narrow feature-size range and homogeneous spatial distribution indicate comparatively uniform nucleation and film growth, with no conspicuous large-scale aggregates or preferentially oriented structures. This morphology is consistent with the predominantly amorphous character identified by XRD.

In contrast, incorporation of Cu(II) produces a substantially more heterogeneous surface (Fig. 2(b)), characterized by irregular aggregates and distinctly elongated features distributed over the film. The apparent lateral dimensions of the larger aggregated features are approximately 0.3–1.2 μm, while the most prominent leaf- or rod-like structure has an estimated width of approximately 0.9–1.0 μm and a length of approximately 4.5–4.7 μm. Thus, compared with the fine-grained morphology of the pristine **PQMHC** film, Cu(II) incorporation promotes the development of larger and anisotropically extended surface features. This evolution is consistent with a change in molecular packing and nucleation kinetics induced by Cu(II) coordination, which can modify intermolecular interactions and favor localized aggregation and directional growth. The resulting increase in morphological heterogeneity provides a plausible structural basis for the subsequent changes in optical behavior through modified light scattering, interfacial electronic pathways, and local electronic disorder^35^.

It should be noted that the above dimensions represent image-derived morphological estimates rather than statistically determined particle-size distributions. Likewise, quantitative surface-roughness parameters such as (R_a_) and (R_q_) cannot be reliably obtained from the present two-dimensional FE-SEM images and require AFM or three-dimensional profilometry. Nevertheless, the calibrated feature dimensions provide direct quantitative evidence supporting the qualitative morphological transition observed upon Cu(II) incorporation.

### Optical analysis

#### Photoluminescence analysis

**Fig. 3:**
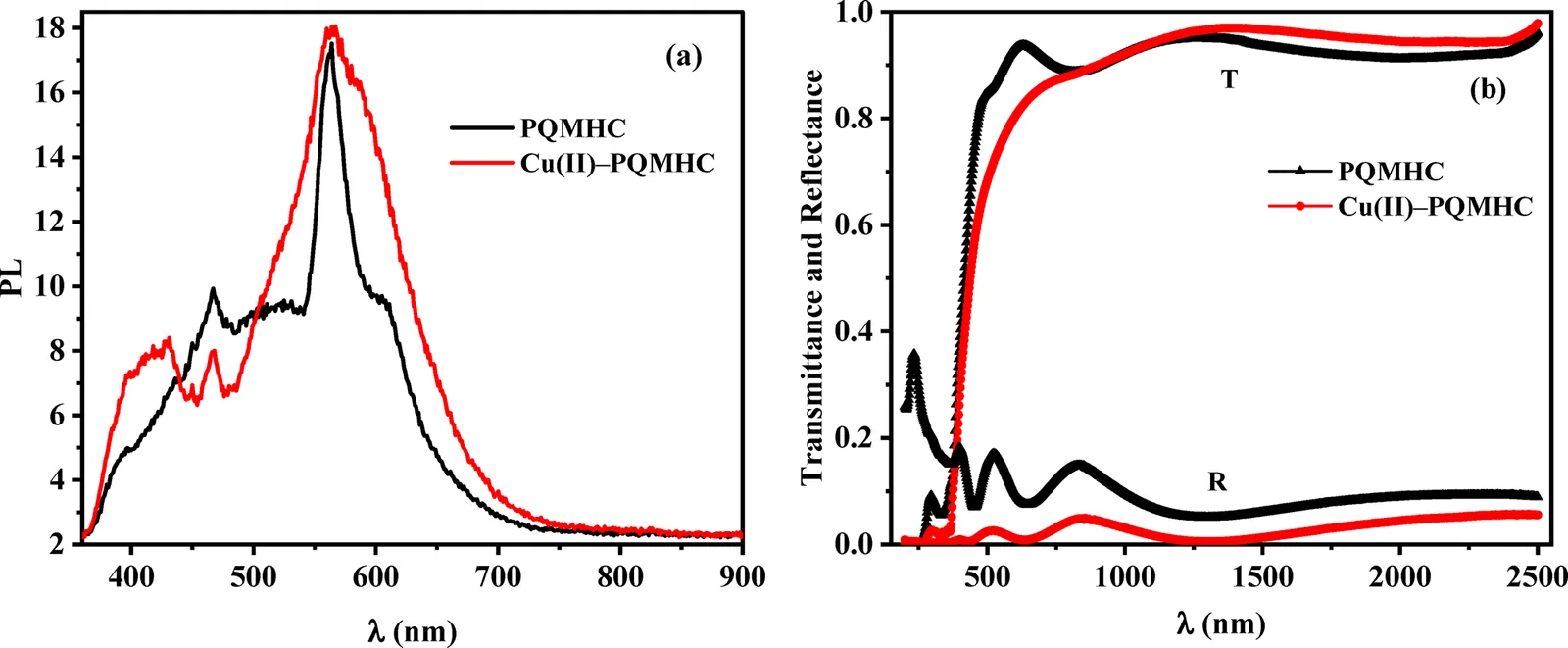
The spectral behavior of (**a**) the photoluminescence (PL), and (**b**) the transmittance (T) and reflectance (R) for **PQMHC** ligand and its Cu(II) complex thin films.

The photoluminescence (PL) spectra of the **PQMHC** ligand and its Cu(II) complex thin films (Fig. 3a) exhibit a broad emission profile spanning the visible spectral region. The emission can be divided into distinct spectral regions: the violet–blue region (~ 380–480 nm), the blue–green region (~ 480–550 nm), and the green–yellow region (~ 550–650 nm).

For the **PQMHC** ligand, weak shoulders appear in the violet–blue region, indicating higher-energy π–π* transitions and localized excited states, and a weak peak located in the blue region is commonly associated with n–π* transitions and shallow defect states. These states arise from structural imperfections or localized lone-pair electrons (e.g., from N or O atoms in the ligand). The dominant emission band is centered in the blue–green region, typically around ~ 500–520 nm, corresponding to greenish emission, which reflects recombination from moderately localized states within the disordered structure.

Upon complexation with Cu(II), the PL spectrum undergoes noticeable changes in both intensity and spectral position. The main emission band is slightly shifted toward longer wavelengths, appearing in the green–yellow region (~ 520–560 nm), indicating a red shift associated with metal–ligand charge transfer (MLCT) interactions and a reduction in the optical band gap. Additionally, the emission intensity is significantly quenched, especially in the blue region, due to enhanced non-radiative recombination pathways introduced by the Cu(II) ions. The broadening of the emission band in the complex further suggests increased structural disorder and a wider distribution of emissive states.

These finding revealed that, the **PQMHC** ligand exhibits relatively stronger emission with identifiable contributions across the violet–blue and blue–green regions, while the Cu(II) complex shows a broader, red-shifted emission extending into the green–yellow region with reduced intensity. These spectral modifications confirm that Cu(II) incorporation alters the electronic structure, promotes non-radiative decay, and shifts the emission toward lower energies, which is crucial for tuning the optical response in optoelectronic applications^36^.

#### Transmittance and reflectance spectrum analysis

Figure 3(b) presents the spectral dependence of the transmittance (T) and reflectance (R) of the **PQMHC** ligand and its Cu(II) complex thin films in the wavelength range of 200–2500 nm. Pronounced changes in the optical response are observed upon Cu(II) coordination, indicating an influence of complexation on the electronic structure of the material.

The transmittance spectrum of the **PQMHC** ligand thin film exhibits high transparency throughout the visible and near-infrared regions, with T values approaching unity above the absorption edge. This behavior suggests weak absorption and low optical losses, characteristic of organic ligand films with π–π* and n–π* electronic transitions confined mainly to the ultraviolet region. The sharp increase in transmittance at shorter wavelengths corresponds to the fundamental absorption edge, reflecting the intrinsic electronic band structure of the ligand.

In contrast, the Cu(II)–**PQMHC** complex demonstrates a noticeable reduction in transmittance over the entire spectral range, particularly in the UV–visible region. This decrease in transparency can be attributed to the formation of additional electronic states induced by metal–ligand coordination. The interaction between the Cu(II) 3d orbitals and the ligand molecular orbitals leads to metal–ligand charge transfer (MLCT) and d–d transitions, thereby enhancing light absorption. Furthermore, the slight red shift of the absorption edge observed for the Cu(II) complex indicates a lowering of transition energies, which is commonly associated with a reduction in the optical band gap due to increased electronic delocalization after complex formation. These findings confirm that Cu(II) complexation significantly modifies the optical absorption behavior of the **PQMHC** ligand.

The reflectance spectra provide complementary insight into the optical properties of the films. The **PQMHC** ligand film exhibits relatively low reflectance with a smooth spectral profile, implying a modest refractive index and minimal surface or bulk scattering. This behavior is consistent with a homogeneous thin film and weak electronic polarization.

Upon Cu(II) complexation, the reflectance increases across the investigated wavelength range, accompanied by the appearance of additional spectral features. The enhanced reflectance can be associated with an increase in the real part of the refractive index and greater polarizability of the complex film. The presence of the Cu(II) ions increases electron density and strengthens the interaction between the incident electromagnetic field and the material. The correlation between reflectance features and transmittance suppression further supports the occurrence of additional absorption mechanisms induced by metal coordination.

Overall, the combined decrease in transmittance and increase in reflectance upon Cu(II) complexation demonstrate a substantial alteration in the optical response of **PQMHC** thin films. These changes arise from modified electronic transitions, increased polarizability, and enhanced light–matter interaction, confirming successful Cu(II) chelation. Such tunability of optical properties highlights the potential applicability of the Cu(II)–**PQMHC** complex in optoelectronic, sensing, and photonic devices where controlled absorption and refractive properties are required.

#### Absorption parameters analysis

**Fig. 4:**
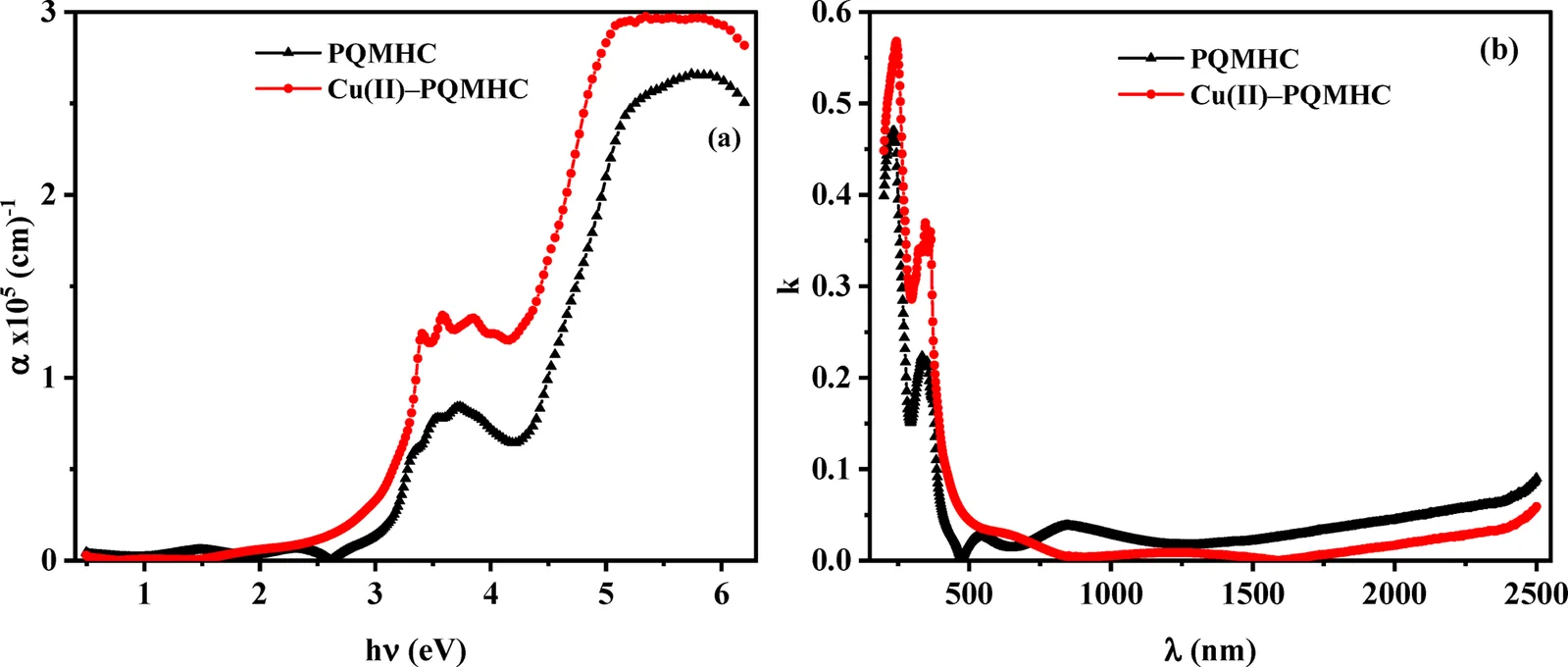
(**a**) The variation of the absorption coefficient (α) with photon energy, and (**b**) the spectral behavior of the absorption index (*k*) of **PQMHC** ligand and its Cu(II) complex thin films.

Figure 4(a) illustrates the variation of the absorption coefficient (α) as a function of photon energy (hν) of **PQMHC** ligand and its Cu(II) complex thin films. As shown in Fig. 4(a), both films exhibit very low absorption coefficients at photon energies below ~ 2.5 eV, indicating weak absorption in the near-infrared and visible regions. A pronounced increase in α is observed beyond this energy, corresponding to the onset of fundamental electronic transitions.

For the **PQMHC** ligand film, the absorption coefficient rises sharply at photon energies above ~ 3.0 eV, reaching values on the order of (2.5–2.7) × 10⁵ cm⁻¹ in the high-energy region near 5.5–6.0 eV. This behavior is characteristic of π–π^*^ and n–π^*^ transitions associated with the conjugated molecular structure of the ligand. The relatively abrupt increase in α reflects the allowed nature of these electronic transitions and indicates a well-defined absorption edge.

In contrast, the Cu(II)–**PQMHC** complex exhibits significantly higher α values across the entire energy range. The absorption onset for the complex occurs at slightly lower photon energies, around ~ 2.8–2.9 eV, followed by a steeper rise in α compared to the free ligand. At photon energies above ~ 5.0 eV, the absorption coefficient of the complex approaches ~ 3.0 × 10⁵ cm⁻¹, exceeding that of the ligand film. This enhancement can be attributed to additional absorption channels introduced by Cu(II) coordination, including metal–ligand charge transfer transitions and Cu(II) d–d transitions. The shift of the absorption edge toward lower photon energies further suggests a reduction in the optical band gap due to increased electronic delocalization upon complex formation.

Overall, the higher absorption coefficient of the Cu(II)–**PQMHC** complex confirms photon absorption and indicates increased oscillator strength resulting from metal–ligand interaction.

The spectral variation of the absorption index (*k*), shown in Fig. 4(b), provides complementary information regarding the attenuation of electromagnetic waves within the films. Both samples display relatively high *k* values in the ultraviolet region (λ ≈ 350–450 nm), where strong electronic absorption occurs.

For the **PQMHC** ligand film, *k* reaches values of approximately 0.40–0.45 near ~ 380–400 nm, followed by a rapid decrease with increasing wavelength. Beyond ~ 500–600 nm, *k* drops to very low values (< 0.05) and remains nearly constant throughout the visible and near-infrared regions, indicating weak absorption and confirming the high transparency observed in the transmittance spectra.

The Cu(II)–**PQMHC** complex exhibits noticeably higher k values in the UV region, reaching up to ~ 0.55–0.58 below ~ 400 nm, which reflects enhanced absorption due to Cu(II)-related electronic transitions. Although k decreases sharply beyond ~ 450–500 nm, it remains slightly higher than that of the ligand throughout the visible and near-infrared regions up to 2500 nm. This behavior signifies persistent absorption contributions from localized states introduced by complexation, even at longer wavelengths.

The combined increase in absorption coefficient and absorption index upon Cu(II) coordination demonstrates a clear enhancement of light–matter interaction in the **PQMHC** thin films. The lower-energy absorption onset, higher α values (up to ~ 3 × 10⁵ cm⁻¹), and increased k in the UV–visible region confirm that metal–ligand complexation modifies the electronic band structure and introduces additional optically active transitions. These findings are consistent with the transmittance and reflectance results and further confirm successful Cu(II) chelation. The enhanced absorption properties of the Cu(II)–**PQMHC** complex highlight its potential applicability in optoelectronic and photonic devices where optical attenuation and tunable electronic structure are required.

#### Energy gap analysis

**Fig. 5:**
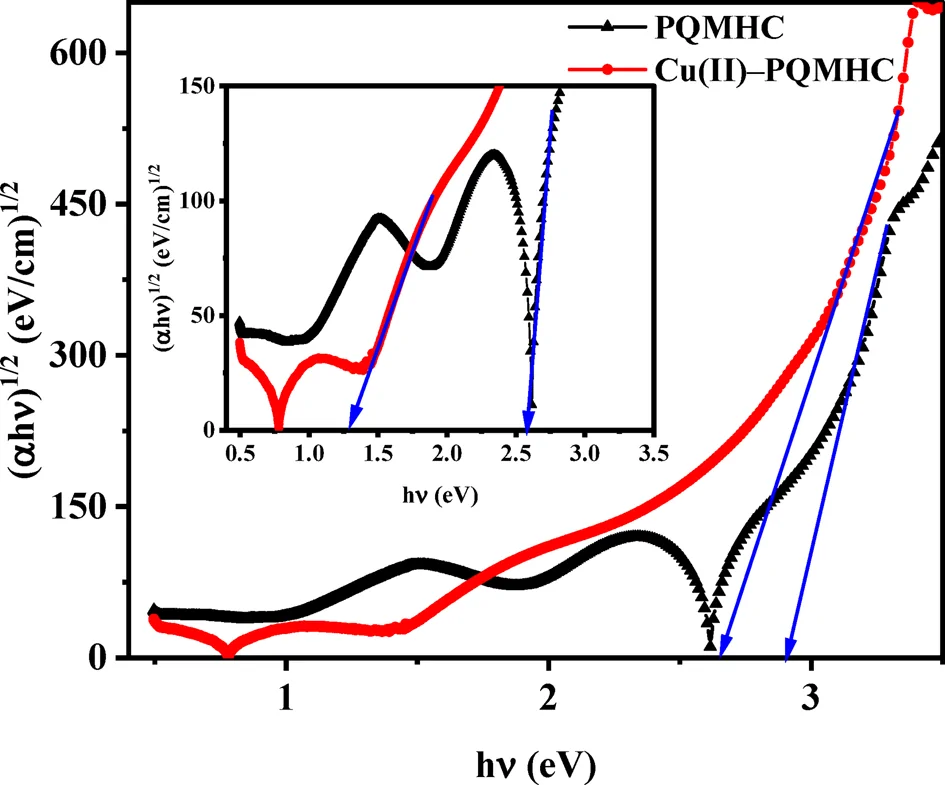
The relation between (αhν)^1/2^ and photon energy (hν) for **PQMHC** ligand and its Cu(II) complex thin films.

Figure 5 depicts the variation of the optical absorption parameter as a function of photon energy (hν) for **PQMHC** and its Cu(II) complex thin films. The well-defined linear regions in the higher-energy range indicate that the dominant optical absorption is associated with indirect transitions. The corresponding optical band-gap values, obtained by extrapolating the linear portions to the photon-energy axis, are listed in Table 1. For the **PQMHC** ligand film, the extrapolated intercept gives E_g_ = 2.87 ± 0.02 eV, consistent with its relatively high visible transmittance and weak absorption. This electronic structure is primarily associated with ligand-centered π–π^*^ and n–π^*^ transitions and the corresponding frontier-orbital separation^37^.

Upon Cu(II) coordination, the absorption edge shifts toward lower photon energies, yielding E_g_ = 2.57 ± 0.01 eV. Although metal–ligand orbital interactions, particularly the coupling between Cu(II) 3d states and ligand orbitals, can contribute to this reduction through the formation and hybridization of additional electronic states, the magnitude of the observed change indicates that other disorder-related mechanisms should also be considered. The low-energy absorption tail evident in the inset of Fig. 5 (Table 1) suggests the presence of localized sub-gap states associated with structural disorder and defect-related transitions, which can broaden the absorption edge^38^.

Notably, the onset gap (E_g_^onset^) decreases much more strongly upon complexation, from 2.54 ± 0.01 to 1.269 ± 0.003 eV, than the principal Tauc gap, which decreases from 2.87 ± 0.02 to 2.57 ± 0.01 eV (Table 1). This disproportionate reduction provides strong evidence that the apparent extension of absorption into the sub-gap region cannot be attributed solely to metal–ligand charge-transfer interactions. Instead, it indicates enhanced band-tail formation, commonly associated with an increased distribution of localized electronic states and structural disorder. This interpretation is consistent with the heterogeneous, clustered morphology observed by FE-SEM in Sect.  4.1.2.

Cu(II) coordination may additionally perturb molecular packing and introduce local potential fluctuations, thereby generating defect-associated states and increasing the density of localized states near the band edges. At the same time, coordination-induced orbital overlap and extended conjugation within the chelated framework may enhance π-electron delocalization, facilitating electronic coupling and contributing to the observed red shift of the absorption edge. Thus, the reduced optical gap should be regarded as the combined outcome of Cu(II)–ligand electronic interactions, localized defect states, increased structural disorder and Urbach-type band-tail broadening, together with enhanced electronic delocalization.

Consequently, the Tauc analysis in Fig. 5 demonstrates that Cu(II) complexation systematically modifies the electronic structure and increases the probability of lower-energy indirect transitions, in agreement with the trends observed in the transmittance, reflectance, absorption coefficient, and absorption-index spectra. The resulting band-gap tunability demonstrates the potential of Cu(II)–**PQMHC** thin films for optical and optoelectronic applications requiring controlled absorption-edge engineering.

**Table 1:** Energy gaps, dispersion and nonlinear parameters for **PQMHC** ligand and its Cu(II) complex thin films.

Sample	$\:{\boldsymbol{E}}_{\boldsymbol{g}}^{\boldsymbol{o}\boldsymbol{n}\boldsymbol{s}\boldsymbol{e}\boldsymbol{t}}$ $\:\left(\boldsymbol{e}\boldsymbol{V}\right)$	$\:{\boldsymbol{E}}_{\boldsymbol{g}}^{\boldsymbol{o}\boldsymbol{p}\boldsymbol{t}\boldsymbol{i}\boldsymbol{c}\boldsymbol{a}\boldsymbol{l}}$ $\:\left(\boldsymbol{e}\boldsymbol{V}\right)$	$\:{\boldsymbol{E}}_{\boldsymbol{d}}$ $\:\left(\boldsymbol{e}\boldsymbol{V}\right)$	$\:{\boldsymbol{E}}_{\boldsymbol{o}}$ $\:\left(\boldsymbol{e}\boldsymbol{V}\right)$	$\:{\boldsymbol{\varepsilon\:}}_{\mathbf{\infty\:}}$	$\:{\boldsymbol{n}}_{0}$	$\:{\boldsymbol{\epsilon\:}}_{\boldsymbol{L}}$	$\:\boldsymbol{N}/{\boldsymbol{m}}^{\boldsymbol{*}}$ $\:\left({\mathrm{kg}}^{-1}{\mathrm{m}}^{-3}\right)$	$\:{\boldsymbol{\chi\:}}^{\left(1\right)}$	$\:{\boldsymbol{\chi\:}}^{\left(3\right)}$ $\:(\mathrm{esu}\mathrm{)}$	$\:{\boldsymbol{n}}_{2}$ $\:(\mathrm{esu}\mathrm{)}$	$\:{\boldsymbol{\chi\:}}_{\mathrm{opt}}$
PQMHC	2.54 ± 0.01	2.87 ± 0.02	3.41	2.37	2.44	1.56	2.83	9.72 × 10^55^	0.114	2.86 × 10⁻¹⁴	6.91 × 10⁻¹³	2.01
Cu(II)–PQMHC	1.269 ± 0.003	2.57 ± 0.01	2.11	2.48	1.85	1.36	2.07	5.61 × 10^55^	0.068	3.63 × 10⁻¹⁵	1.01 × 10⁻¹³	2.08

#### Dispersion parameters analysis

**Fig. 6:**
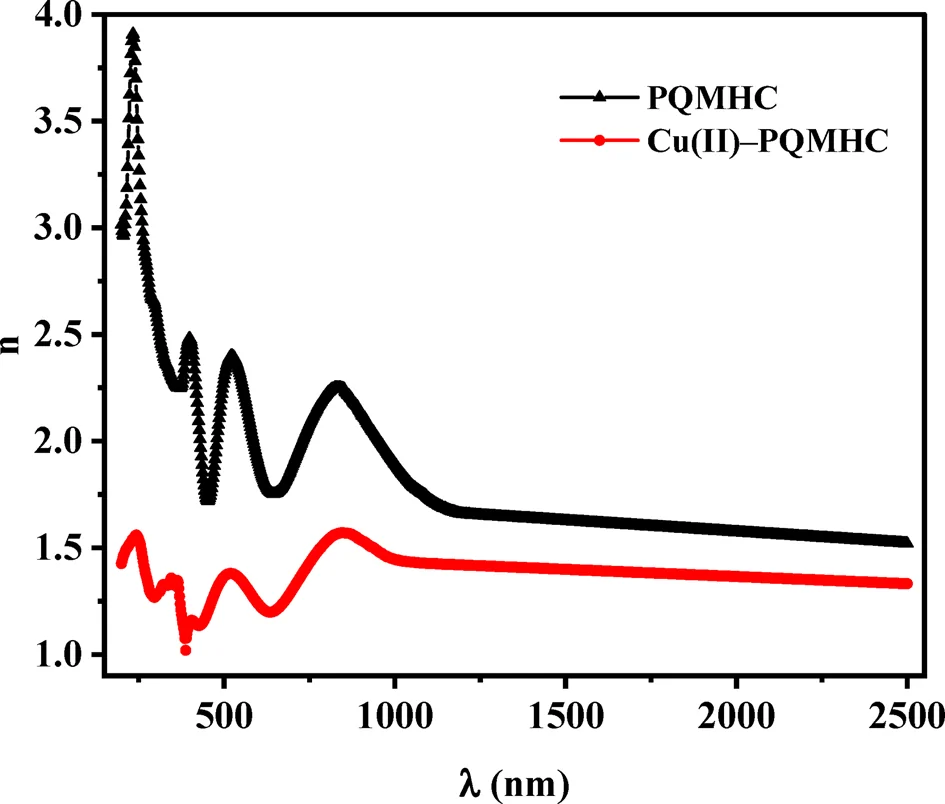
The spectral behavior of refractive index (*n*) for **PQMHC** ligand and its Cu(II) complex thin films.

Figure 6 presents the spectral variation of the refractive index (*n*) of **PQMHC** ligand and Cu(II)–**PQMHC** complex thin films in the wavelength range of ~ 200–2500 nm. Both films exhibit typical anomalous dispersion behavior, with higher refractive index values in the ultraviolet region followed by a gradual decrease with increasing wavelength as normal dispersion dominates.

The **PQMHC** ligand film shows relatively high refractive index values in the UV region, reaching ~ 3.8 below 450 nm, due to strong electronic polarization near the absorption edge. With increasing wavelength, *n* decreases sharply and attains an almost constant value of ~ 1.5 in the near-infrared region, indicating normal dispersion and reduced absorption losses^39,40^.

The Cu(II)–**PQMHC** complex exhibits lower refractive index values over the entire spectral range, with n varying from ~ 1.6 in the UV to ~ 1.3 in the near-infrared region. The reduced magnitude and smoother dispersion profile of n upon Cu(II) complexation indicate modified electronic polarizability and structural rearrangement induced by metal–ligand coordination.

Overall, the decrease in refractive index after Cu(II) complexation confirms that metal coordination effectively alters the dispersion characteristics of **PQMHC** thin films, enabling the tunability of their refractive properties for optical and optoelectronic applications.

**Fig. 7:**
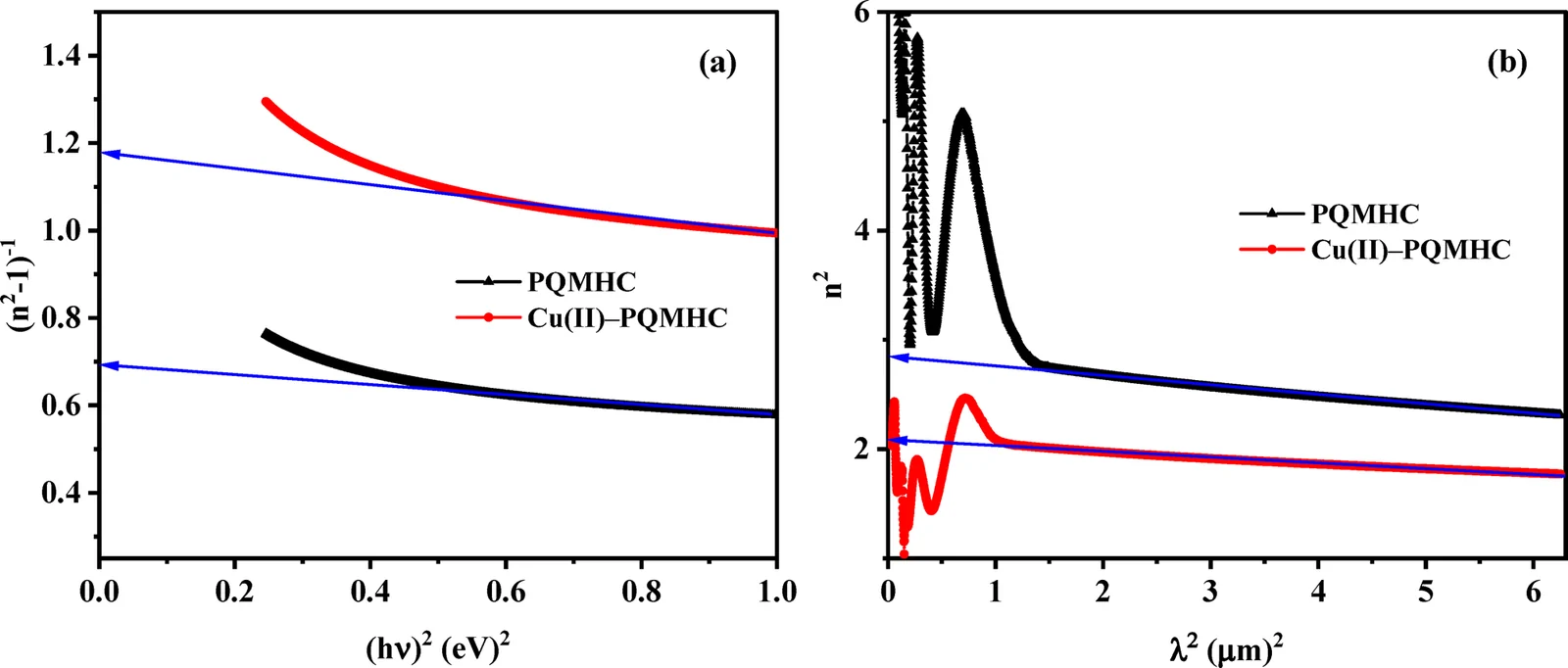
The relation of (**a**) (n^2^ − 1)^−1^ with (hν)^2^, and (**b**) n^2^ with λ^2^ for the **PQMHC** ligand and its Cu(II) complex thin films.

The dispersion behavior of the refractive index for the **PQMHC** ligand and its Cu(II) complex thin films was analyzed using the Wemple–DiDomenico single-oscillator model^41^. The good linearity observed in the plot of $\:\left({n}^{2}-1{)}^{-1}\right.$versus $\:\left(h\nu\:{)}^{2}\right.$in Fig. 7(a) validates the applicability of the model, and the extracted optical dispersion parameters are summarized in Table 1. From the linear fits, the oscillator energy ($\:{E}_{o}$) and dispersion energy ($\:{E}_{d}$) were determined. For the **PQMHC** ligand, $\:{E}_{o}=2.37$eV and $\:{E}_{d}=3.41$eV, while for the Cu(II) complex, $\:{E}_{o}$ slightly increased to 2.48 eV and $\:{E}_{d}$ decreased to 2.11 eV. The higher $\:{E}_{d}$ value for the ligand indicates stronger interband optical transitions and a relatively more ordered electronic structure, whereas the reduction in $\:{E}_{d}$ upon complexation suggests weakened transition strength due to increased structural disorder and modification of the electronic environment induced by Cu(II) coordination.

Additionally, the high-frequency dielectric constant ($\:{\varepsilon\:}_{{\infty\:}}$) and static refractive index ($\:{n}_{0}$), $\:{\varepsilon\:}_{{\infty\:}}={n}_{0}^{2}$^42^ were evaluated, yielding values of 2.44 and 1.56 for the **PQMHC** ligand, and 1.85 and 1.36 for the Cu(II) complex, respectively. The decrease in both $\:{\varepsilon\:}_{{\infty\:}}$ and $\:{n}_{0}$ for the complex reflects reduced electronic polarizability, which can be attributed to electron density redistribution and altered intermolecular interactions following metal incorporation. Overall, these results demonstrate that Cu(II) complexation significantly modifies the optical dispersion characteristics and electronic structure of **PQMHC**-based thin films. These findings suggest that the Cu(II)–**PQMHC** complex thin films may be promising for optoelectronic applications such as optical coatings, waveguides, and photonic devices, where controlled refractive index and dielectric properties are essential.

The refractive index dispersion at longer wavelengths was further analyzed using the relation between $\:{n}^{2}$and $\:{\lambda\:}^{2}$. The linear variation of $\:{n}^{2}$as a function of $\:{\lambda\:}^{2}$, presented in Fig. 7(b), confirms the validity of this approach, and the extracted parameters are listed in Table 1. The lattice dielectric constant ($\:{\varepsilon\:}_{L}$) was obtained from the intercept of the linear plots, yielding values of 2.83 and 2.07 for the **PQMHC** ligand and its Cu(II) complex, respectively. These values are higher than the corresponding high-frequency dielectric constants ($\:{\epsilon\:}_{{\infty\:}}$), reflecting the additional contribution of lattice polarization at longer wavelengths.

The **PQMHC** ligand exhibits a larger difference between $\:{\varepsilon\:}_{L}$ and $\:{\varepsilon\:}_{{\infty\:}}$, indicating stronger lattice polarization and higher dispersion contribution, which reflects enhanced interaction of bound charges with the applied field. In contrast, the Cu(II) complex shows reduced $\:{\epsilon\:}_{L}$ and $\:{\varepsilon\:}_{{\infty\:}}$ values with a smaller separation between them, suggesting decreased polarizability and weakened lattice contribution due to metal coordination. This behavior is consistent with dispersion-dependent optical response, where reduced separation between dielectric constants corresponds to diminished electronic and ionic polarization, as reported in similar optoelectronic systems in the literature^43^.

The carrier concentration to effective mass ratio ($\:N/{m}^{*}$) was also evaluated from the slope and found to decrease from $\:9.72\times\:{10}^{55}$ to $\:5.61\times\:{10}^{55}{\text{}\mathrm{kg}}^{-1}{\mathrm{m}}^{-3}$ upon complexation. This reduction indicates a decrease in free carrier contribution and enhanced carrier localization, which can be attributed to structural disorder and electronic modification induced by Cu(II) coordination^44^. Overall, these results are consistent with the Wemple–DiDomenico analysis and further confirm that metal complexation significantly influences the dielectric response and electronic structure of **PQMHC**-based thin films.

#### Dielectric parameters analysis

The suitability of a material for optoelectronic applications can be assessed through its dielectric constant, which describes its response to an external electric field. In solids, polarization is directly related to the dielectric constant, while the dielectric response generally decreases with increasing field strength. The complex dielectric constant provides insight into optical behavior: the real part governs electromagnetic wave propagation and the reduction of light velocity in the medium, whereas the imaginary part represents energy dissipation due to absorption and relaxation processes^45^.

**Fig. 8:**
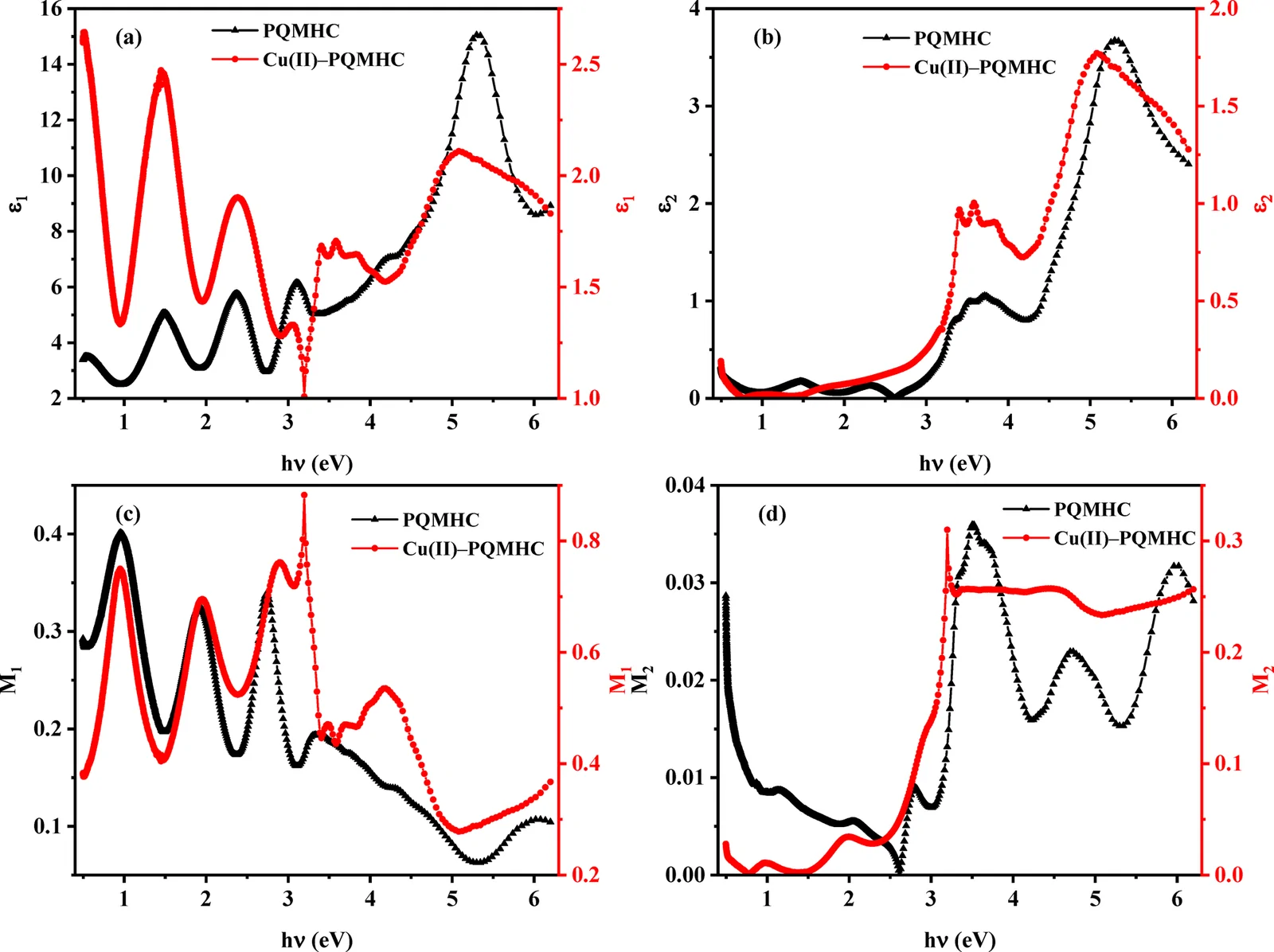
The relation between (**a**) * \varepsilon *_1_, (**b**) * \varepsilon *_2_, (**c**) M_1_, and (**d**) M_2_ with hν for **PQMHC** ligand and its Cu(II) complex thin films.

Figure 8(a) shows the dependence of the real part of the dielectric constant ($\:{\varepsilon\:}_{1}$) on photon energy. For both **PQMHC** and its Cu(II) complex thin films, $\:{\varepsilon\:}_{1}$exhibits pronounced dispersion in the low-energy region, characterized by several oscillatory features. These oscillations arise from resonance effects associated with electronic polarization and bound charge carrier response. The Cu(II)–**PQMHC** complex exhibits higher $\:{\varepsilon\:}_{1}$ values compared to the pristine **PQMHC** ligand over most of the investigated energy range. This enhancement indicates increased polarizability and dielectric strength due to coordination between Cu(II) ions and the **PQMHC** ligand, which modifies the local electronic environment. At higher photon energy, the increase in $\:{\varepsilon\:}_{1}$ reflects the contribution of interband transitions, followed by a gradual reduction as polarization mechanisms approach saturation^46^.

The imaginary part of the dielectric constant ($\:{ \varepsilon\:}_{2}$), shown in Fig. 8(b), represents dielectric loss and optical absorption behavior. In the low-energy region, $\:{\varepsilon\:}_{2}$ values are minimal for both samples, corresponding to weak absorption. Beyond ~ 3 eV, a sharp rise in $\:{\varepsilon\:}_{2}$ is observed, indicating the onset of allowed interband electronic transitions. The Cu(II)–**PQMHC** complex displays higher $\:{\varepsilon\:}_{2}$ values and broader absorption features than the **PQMHC** ligand, confirming enhanced optical absorption due to metal–ligand charge transfer and d–π* electronic interactions. This behavior demonstrates that complex formation introduces additional electronic states, increasing transition probability and dielectric loss^47^.

A powerful parameter for probing relaxation mechanisms is the complex electric modulus, which emphasizes bulk relaxation processes while minimizing electrode polarization effects^27^.

Figure 8(c) illustrates the variation of the real part of the electric modulus ($\:{M}_{1}$) with photon energy. The $\:{M}_{1}$ values decrease gradually with increasing energy after exhibiting fluctuations in the low-energy region. These features indicate the progressive suppression of electrode polarization effects and enhanced bulk response. The Cu(II)–**PQMHC** complex shows lower $\:{M}_{1}$ values compared to the **PQMHC** ligand, particularly in the low-energy region. This reduction suggests increased dielectric permittivity and improved charge carrier mobility in the complex, reflecting stronger interaction between the electric field and charge carriers due to Cu(II) coordination.

Figure 8(d) presents the imaginary part of the electric modulus ($\:{M}_{2}$), which is highly sensitive to relaxation dynamics within the thin films. At low photon energies, $\:{M}_{2}$ values approach zero, indicating dominance of long-range polarization effects. A pronounced peak appears at higher energies, corresponding to relaxation processes associated with charge carrier hopping and localized motion. The Cu(II)–**PQMHC** complex exhibits a shifted and more intense $\:{M}_{2}$ peak compared to the **PQMHC** ligand, signifying modified relaxation behavior and shorter relaxation times. This behavior reflects enhanced charge transport properties induced by metal–ligand interactions and structural reorganization in the complex.

The combined dielectric constant and electric modulus analysis reveals that complexation of **PQMHC** with Cu(II) significantly alters polarization, absorption, and relaxation mechanisms in thin films. The increased $\:{ \varepsilon \:}_{1}$ and $\:{ \varepsilon\:}_{2}$, along with reduced $\:{M}_{1}$ and enhanced $\:{M}_{2}$, confirm improved optical activity and charge carrier dynamics in the Cu(II)–**PQMHC** complex. These features highlight the potential of the complex for advanced optoelectronic and photonic applications in the future.

**Fig. 9:**
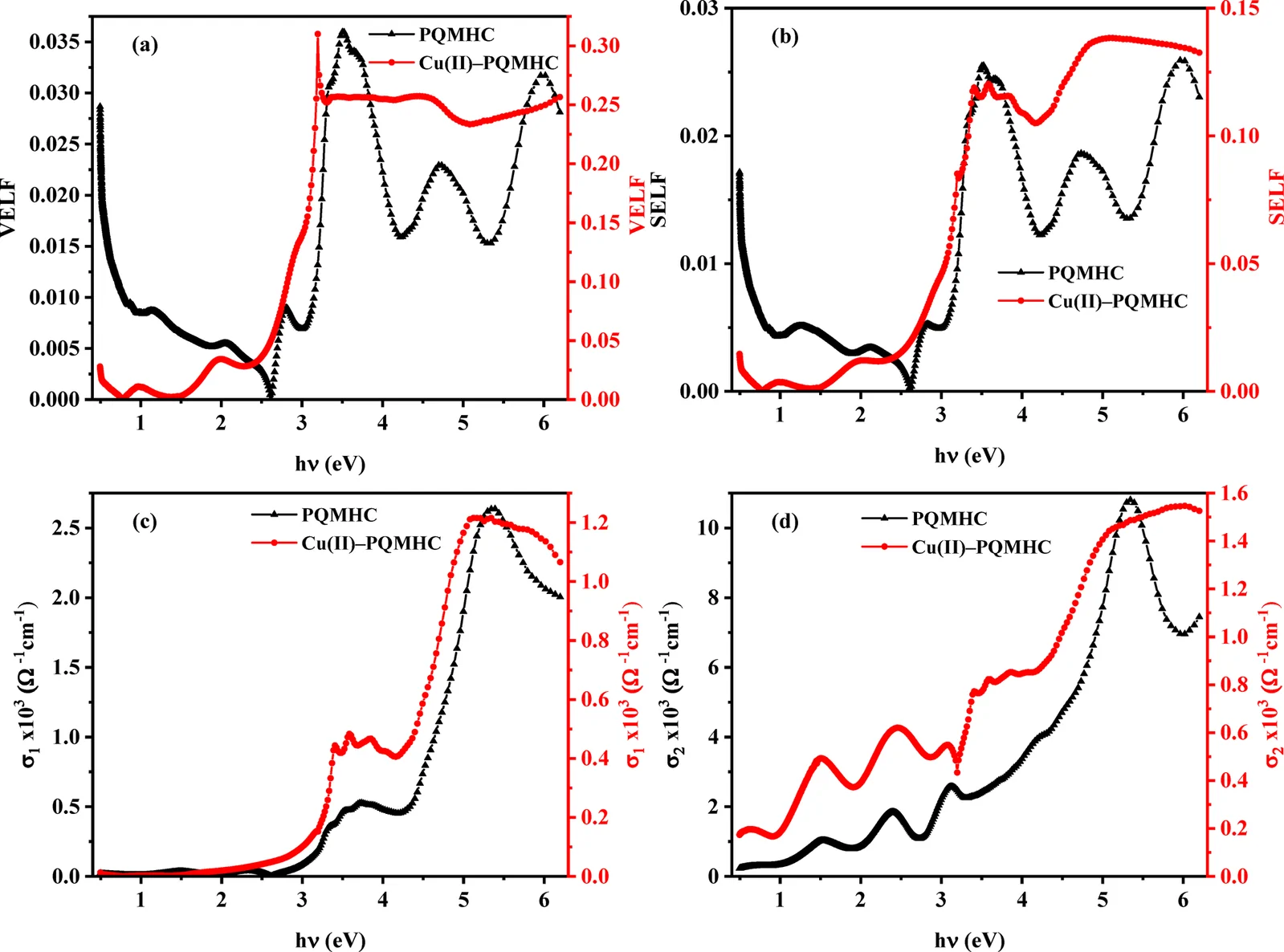
The relation between (**a**) VELF, (**b**) SELF, (**c**) σ_1_, and (**d**) σ_2_ with hν for **PQMHC** ligand and its Cu(II) complex thin films.

Figure 9(a) depicts the dependence of the volume energy loss function (VELF) on photon energy for the **PQMHC** ligand and its Cu(II)–**PQMHC** complex thin films. VELF represents the energy loss of fast electrons traversing the bulk of the material and is highly sensitive to plasma resonance conditions. For both samples, VELF exhibits low values at lower photon energies, indicating weak bulk plasmon excitation^48^. A pronounced peak appears in the higher energy range (~ 3–4 eV), corresponding to volume plasmon resonance, where collective oscillations of charge carriers are dominant. The Cu(II)–**PQMHC** complex shows higher VELF values and a broader resonance peak compared to the **PQMHC** ligand, reflecting enhanced bulk energy loss caused by increased free-carrier density and stronger electronic interactions induced by Cu(II) coordination^49^.

The surface energy loss function (SELF), shown in Fig. 9(b), describes the energy loss of electrons at the surface of the thin films. Similar to VELF, SELF values are minimal at low photon energies, signifying negligible surface plasmon excitation. With increasing photon energy, SELF rises sharply beyond ~ 3 eV and exhibits distinct peaks associated with surface plasmon resonances. The Cu(II)–**PQMHC** complex exhibits higher SELF values compared to the ligand, confirming increased surface-related energy dissipation. This behavior suggests stronger electromagnetic field confinement at the surface and improved surface electronic activity due to metal–ligand interactions.

Figure 9(c) presents the variation of the real part of optical conductivity ($\:{\sigma\:}_{1}$) as a function of photon energy. $\:{\sigma\:}_{1}$ reflects the contribution of free and bound charge carriers to charge transport and optical absorption. At lower photon energies, $\:{\sigma\:}_{1}$ values remain relatively low, indicating limited carrier excitation^50^. A significant increase in $\:{\sigma\:}_{1}$ is observed beyond ~ 3 eV, corresponding to the onset of interband transitions. The Cu(II)–**PQMHC** complex exhibits higher $\:{\sigma\:}_{1}$ values than **PQMHC**, demonstrating enhanced carrier density and improved charge transport due to the introduction of additional electronic states by Cu(II) complexation.

The imaginary part of optical conductivity ($\:{\sigma\:}_{2}$), shown in Fig. 9(d), is associated with polarization effects and reactive charge carrier response. $\:{\sigma\:}_{2}$ increases gradually with photon energy and shows pronounced features in the higher energy region, reflecting strong polarization associated with electronic transitions. The Cu(II)–**PQMHC** complex displays consistently higher $\:{\sigma\:}_{2}$ values and smoother dispersion compared to the ligand. This enhancement indicates stronger capacitive behavior and increased energy storage capability, arising from enhanced electron delocalization and metal–ligand charge transfer mechanisms.

The energy loss and optical conductivity analyses reveal that the incorporation of Cu(II) ions into the **PQMHC** ligand substantially modifies both bulk and surface electronic responses. The enhanced VELF and SELF indicate stronger plasmonic excitations, while the increased $\:{\sigma\:}_{1}$ and $\:{\sigma\:}_{2}$ values confirm improved charge transport and polarization behavior. These findings suggest that Cu(II)–**PQMHC** complex thin films have modified optoelectronic characteristics relative to the ligand, indicating potential suitability for plasmonic, photonic, and optoelectronic device applications.

#### Estimated non-linear optical parameters

The nonlinear optical parameters of **PQMHC** and its Cu(II) complex thin films listed in Table 1 were estimated using Miller’s empirical rule^51^ from the corresponding linear optical parameters. It is important to emphasize that these values represent theoretical estimates rather than direct experimental measurements of nonlinear optical response. Miller’s rule provides a useful dispersion-based approach for assessing relative nonlinear optical trends; however, the calculated parameters should not be interpreted as definitive experimental nonlinear susceptibilities. Direct techniques, such as Z-scan measurements, would be required to quantitatively determine the nonlinear absorption and nonlinear refractive response of the films.

The calculated linear optical susceptibility, χ⁽¹⁾, is 0.114 for the **PQMHC** ligand and 0.068 for the Cu(II) complex, indicating a comparatively higher calculated linear polarizability for the ligand. Correspondingly, the estimated third-order nonlinear susceptibility, χ⁽³⁾, decreases from $\:2.86\times\:{10}^{-14}$ esu for **PQMHC** to $\:3.63\times\:{10}^{-15}$ esu for the Cu(II) complex, while the calculated nonlinear refractive index, n₂, exhibits a similar decrease from $\:6.91\times\:{10}^{-13}$ esu to $\:1.01\times\:{10}^{-13}$ esu, respectively (Table 1). These results suggest a lower predicted nonlinear response following Cu(II) coordination. Rather than being regarded as direct evidence of suppressed nonlinear optical performance, this trend is interpreted as an indication of reduced calculated polarizability within the Miller-rule framework. The change may be associated with Cu(II)-induced modification of π-electron delocalization, molecular conjugation, and charge-transfer pathways.

This interpretation is broadly consistent with Priyadarshini et al.^52^ and Das et al.^53^, who demonstrated that structural and compositional modifications can substantially alter dispersion and related optical parameters in thin-film and heterostructured materials. Conversely, enhancement of nonlinear optical parameters has also been reported in conjugated and interface-engineered systems, where interfacial charge transfer, defect-related polarization centers, or local-field effects can increase the nonlinear response^54^. Such differences emphasize that the direction and magnitude of nonlinear optical changes depend strongly on the specific structural and electronic modifications introduced by material design. In the present system, the calculated decrease may be related to Cu(II)-induced changes in conjugation and electronic delocalization; however, this interpretation remains a proposed mechanism rather than a direct experimental determination.

Furthermore, the calculated optical electronegativity increases slightly from 2.01 for **PQMHC** to 2.08 for the Cu(II) complex, suggesting a modest increase in electron-attracting tendency following coordination. This change is qualitatively consistent with the reduction in the calculated polarizability and Miller-rule-derived nonlinear parameters. Overall, the calculated values provide a useful comparative indication of the potential nonlinear optical trends of the two films and establish a basis for selecting the more promising composition for future nonlinear-optical studies. Nevertheless, quantitative claims regarding nonlinear optical performance are beyond the scope of the present work and require direct experimental validation, particularly through Z-scan or other established nonlinear-optical measurements.

## Conclusion

In this work, **PQMHC** and Cu(II)–**PQMHC** thin films were successfully fabricated via thermal evaporation and systematically investigated to elucidate the influence of metal coordination on their structural, morphological, and optoelectronic properties. XRD analysis confirmed the amorphous nature of both films, indicating the absence of long-range crystalline order, while FE-SEM micrographs revealed a distinct morphological evolution from relatively compact granular features in pristine **PQMHC** to heterogeneous, clustered, and rod-like structures after Cu(II) incorporation. This transformation reflects significant changes in nucleation and growth dynamics as well as enhanced intermolecular interactions induced by metal coordination. Optical characterization demonstrated a clear modulation of the electronic structure upon Cu(II) complexation. The optical band gap decreased from 2.87 ± 0.02 eV for **PQMHC** to 2.57 ± 0.01 eV for the Cu(II) complex, suggesting enhanced electronic delocalization and strengthened charge-transfer interactions between ligand and metal center. In parallel, a reduction in transmittance and an increase in absorption coefficient within the UV–visible region were observed, confirming improved photon absorption and stronger light–matter interaction. Photoluminescence spectra exhibited broad emission bands spanning 380–650 nm, with a noticeable red shift toward 520–560 nm in the Cu(II) complex, further supporting the formation of metal–ligand charge-transfer states and modification of radiative recombination pathways. Dispersion analysis based on the Wemple–DiDomenico single-oscillator model revealed a significant decrease in dispersion energy (E_d_) from 3.41 eV to 2.11 eV and oscillator energy (E_0_) from 2.37 eV to 1.85 eV upon Cu(II) coordination. These changes indicate a substantial alteration in interband transition strength and dielectric polarization behavior, reflecting modified electronic polarizability and band structure characteristics. Consistently, dielectric constant and optical conductivity studies confirmed enhanced polarization response and improved charge-carrier interaction in the Cu(II)-containing system. In contrast, the linear optical susceptibility decreased from 0.114 for **PQMHC** to 0.068 for the Cu(II) complex, indicating a reduction in linear polarizability after coordination. The third-order nonlinear susceptibility χ^(3)^ and nonlinear refractive index n₂ were estimated to decrease from 2.86 × 10⁻¹⁴ esu and 6.91 × 10⁻¹³ esu for the ligand to 3.63 × 10⁻¹⁵ esu and 1.01 × 10⁻¹³ esu for the Cu(II) complex, respectively. However, it is important to emphasize that these nonlinear optical parameters were derived using empirical relations based on refractive index data and therefore represent only approximate trends rather than direct experimental measurements. Consequently, they should not be interpreted as definitive evidence of strong nonlinear optical behavior, and any discussion of NLO performance in this work is limited to qualitative comparative trends. The slight increase in optical electronegativity from 2.01 (**PQMHC**) to 2.08 (Cu(II) complex) further suggests an enhanced electron-withdrawing character upon metal coordination, which is consistent with the observed reduction in polarizability and nonlinear response. Overall, the results establish a comprehensive correlation among structural characteristics, morphological evolution, dielectric response, photoluminescence, and linear optical properties, demonstrating that Cu(II) coordination provides an effective means of modifying the optical response of **PQMHC**-based thin films. The measured and estimated optical parameters suggest that these materials may have potential relevance to optoelectronic, photonic, sensing, and related optical applications; however, these applications should be regarded as prospective rather than demonstrated, since no device was fabricated or subjected to device-level performance evaluation in the present study. Similarly, the nonlinear optical coefficients derived using Miller’s empirical rule represent theoretical estimates and should not be interpreted as direct experimental evidence of nonlinear optical performance. Direct nonlinear optical measurements, such as Z-scan, pump–probe spectroscopy, or optical Kerr-effect measurements, are therefore required to validate these predictions. Future studies should also employ multiple independently prepared samples and statistical analysis to further establish experimental reproducibility. In addition, density functional theory (DFT) calculations, including hyperpolarizability and charge-transfer analyses, together with systematic investigation of alternative metal centers, could provide deeper insight into the electronic origins of the observed optical modifications and guide the rational optimization of **PQMHC**-based materials for future optoelectronic and photonic applications.

## Data Availability

The datasets used and/or analysed during the current study available from the corresponding author on reasonable request.
